# Assessing the application of non-pharmacological interventions for people with dementia in German nursing homes: feasibility and content validity of the dementia care questionnaire (DemCare-Q)

**DOI:** 10.1186/1756-0500-7-950

**Published:** 2014-12-23

**Authors:** Rebecca Palm, Kerstin Köhler, Sabine Bartholomeyczik, Bernhard Holle

**Affiliations:** German Center for Neurodegenerative Diseases (DZNE), Stockumer Str. 12, Witten, 58453 Germany; Faculty of Health, School of Nursing Science, Witten/Herdecke University (UW/H), Stockumer Str. 12, Witten, 58453 Germany

**Keywords:** Questionnaires, Feasibility studies, Health care surveys, Data collection, Non-pharmacological interventions, Nursing home, Dementia

## Abstract

**Background:**

Non-pharmacological interventions are guideline-recommended as initial treatment for people with dementia in nursing homes. In Germany, there is no instrument available to collect standardized data on the application of all of these interventions; an investigation of their use in large-scale samples is not currently possible. This article describes the development and initial testing of a questionnaire (Dementia Care Questionnaire (DemCare-Q)) to assess provided non-pharmacological interventions in residents with dementia in nursing homes that can be completed by nurses.

**Methods:**

The questionnaire development comprised the following steps to achieve content validity and feasibility: a structured content analysis of the German guideline for the care of people with dementia and challenging behavior in nursing homes and a systematic literature review of projects that implemented these; quantitative expert ratings and calculation of content validity indices; qualitative pre-test with future users using cognitive techniques; quantitative pre-test using frequency analysis of item non-response.

**Results:**

The developed questionnaire covers seven dementia-specific non-pharmacological interventions in nursing homes. Problematic items could be improved by revising them successively, bringing forth a feasible and content valid version of the DemCare-Q. The DemCare-Q enables researchers to collect data on the application of dementia interventions in German nursing homes in large-scale studies.

**Conclusion:**

A literature review, expert rating and multi-method pre-test are important steps of questionnaire development. The applied methods ensure content validity and the practicability of the instrument. The publication of this process enhances the transparency of questionnaire design and supports researchers in solving problems in developing questions to assess the application of interventions. Since these are initial steps of questionnaire development, further testing of its reliability is needed.

## Background

Providing care for people with dementia is a great challenge for modern health care systems. By 2050, the expected number of people with dementia worldwide will almost quadruple to 106 million; 43 % of these cases will require continuous care due to the decline of cognitive and functional abilities associated with the progressing disease [1]. In Germany, up to 50% of people with dementia must move into a nursing home in the course of the disease [2]. Due to the rising population of residents with dementia in nursing homes, the provision of care has changed over the last twenty years from a biomedical to a psychosocial perspective [3]. Treatments other than pharmacological ones are in the focus of nursing home providers and consumers. In this respect, the implementation of non-pharmacological interventions as initial treatment is recommended for residents with dementia and challenging behavior [4]. In Germany, recommended interventions are behavioral, pain and cognition assessments, case conferences, multi-sensory stimulation, validation, reminiscence therapy, enhanced physical activities and interventions for managing an acute psychiatric crisis [5].

The monitoring and assessment of nursing home care is a crucial foundation for the development of care strategies and improvement in quality of care. Knowledge regarding the application of recommended interventions is relevant for two reasons:

- From the health service research perspective, it is important to describe the degree of diffusion of interventions in order to establish which of the recommended interventions are used in practice after publication.
- The exploration of associated factors (such as structural or resource conditions of interventions in use) enables researchers to further define and develop the interventions.

These two aspects are the aim of an observational descriptive longitudinal study in German nursing homes (DemenzMonitor) [6]. The DemenzMonitor is designed as a large-scale study with annual data collections. For this purpose, an instrument is needed that allows assessment of the application of recommended dementia specific interventions and is shown to be valid and feasible under research conditions in German nursing homes.

By and large, there are three possible data sources: residents’ records, staff observations and self-reporting. Residents’ records provide extensive information but are often difficult to obtain and may lack accuracy, completeness and validity [7]. Standardized observational methods can also provide valuable information on care practices but require intense resources (e.g. Dementia Care Mapping) [8, 9]. Given these constraints, self-reported data were considered more feasible for large-scale and multi-center studies. Self-reported data can be derived from either the residents or the staff. Because the accuracy of self-reported data from residents with dementia can be considered questionable [7], we decided to use a care staff self-report questionnaire. This questionnaire should be completed by nursing home staff with a small amount of prior training and supervision to enable data collections in large samples.

Assessment scales that allow a data collection on applied nursing interventions in dementia care that can be used by nursing staff in large-scale studies are rare. The most common instrument that is implemented in many countries worldwide is the Resident Assessment Instrument (RAI) for long term care facilities in version 2.0. This instrument is intended to be completed by nurses and has shown satisfactory validity in research [10, 11]. The main focus of the RAI 2.0 is assessment of resident resources, but it also assesses the application of special treatments and procedures with respect to dementia care (e.g., special care for residents with dementia, intervention programs for mood, behavior and cognitive loss). Hereby, it does not specify the characteristics of these interventions (e.g., type of special care, duration and frequency of special programs). Therefore, a detailed assessment of the application of recommended dementia specific interventions is not possible with the RAI. Another instrument considered with regard to the research aim of the DemenzMonitor was developed by Wingenfeld and colleagues in 2011 in Germany [12]. Nurses can also complete this instrument, but it primarily aims at measuring health-related outcomes, for use within the mandatory external quality assurance process and not for research purposes. Providing that this instrument might be implemented in Germany, its scientific use needs to be evaluated to use it for research purposes. The instrument covers the full range of care-related health conditions, including some aspects of dementia care (assessment of pain and behavior, physical activity) but misses other aspects of interest such as the provision of case conferences, validation, multisensory stimulation and reminiscence therapy. To our knowledge, no instrument fulfills the described requirements for the use in the DemenzMonitor study, so we decided to develop a new instrument.

The development of a valid tool to measure the application of nursing interventions in practice comprises several steps and revisions [13, 14]. We consider the sound investigation of the utility, clarity and accuracy as a prerequisite for pilot testing and further revision and testing. Therefore, we aim to present the development and initial validity assessment of the questionnaire called the “Dementia Care Questionnaire” (DemCare-Q). Based on relevant nursing literature we developed an initial set of items and applied qualitative and quantitative methods to test for content validity and feasibility. Different revision steps followed the analysis.

## Methods

**Figure 1:**
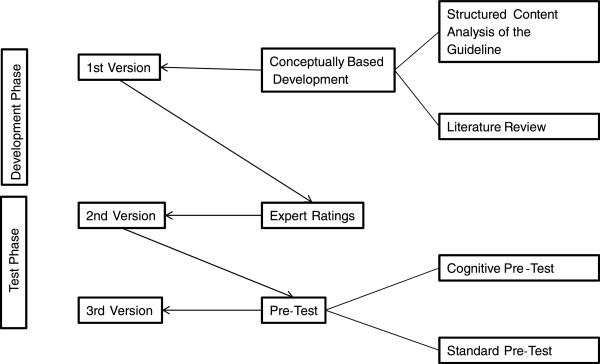
**Process of questionnaire development and testing.**

The questionnaire was developed in two phases (development and test phase) that involved different methodological steps: conceptually based development, expert ratings, and a pre-test (Figure 1).

The ethical committee of the German Society of Nursing Science approved the study protocol of the test procedures, information letters, and consent forms. All participants (nurses, residents resp. their legal guardians, experts) were informed about the aims of the study and asked to give written consent.

### Step 1: conceptual development

The item development refers to the German guidelines for the care of people with dementia and challenging behavior in nursing homes [5]. The research team (RP, KK) analyzed the guidelines and cited international publications for general definitions and descriptions of the interventions by conducting a structured content analysis [15]. For the coding procedure, the software MaxQDA (version 11) was used. As a result, a criteria catalogue was developed that summarized information on the theoretical foundation, frequency and duration of application; the qualifications needed to apply the intervention; and adaptations based on dementia severity. Additionally a systematic literature review was conducted to deepen knowledge about practice variations and to validate the criteria catalogue [16]. Based on knowledge from the literature we compiled an initial set of items (version 1 of the DemCare-Q).

### Step 2: expert ratings

Version 1 of the DemCare-Q was rated by content experts following a standardized procedure.

### Study participants

Eight participants from Germany were selected because of their expelled expertise in the field of dementia care in German nursing homes. The experts were recruited by a search of relevant databases, web-sites, and networks and were contacted by personal letter.

Six of the participants were trained nurses, of whom four were working in research. One nurse was working in a leading position in a nursing home, and one as a self-employed trainer for validation therapy. The other two experts were a psychologist and a physician, both of whom worked in the field of dementia research at the time. Six of these experts were specifically trained in dementia care. Their practical experience ranged from 2 years to 24 years, with a median of 8 years. The researchers had a mean work experience of 11 years, ranging from 5 to 20 years.

The experts were given explanations about the aim of the DemenzMonitor study, a consent form, written instructions, the rating instrument, and a questionnaire assessing their expertise in the field.

### Testing procedure of the standardized expert rating

The experts were asked to rate

- The relevance and comprehensibility of the questions,
- The completeness and reasonability of the answers.

The ratings were performed using a four –point –rating -scale [17, 18]. An item-level score of 1 or 2 indicated low relevance or a need for major revision because of low comprehensibility, completeness or reasonability. An item -level score of 3 or 4 indicated acceptable content validity.

Two rounds of expert ratings were conducted within a time period of two months. Between the two rounds, the instrument was revised. In the second round, the same group of experts was asked solely to judge the comprehensibility, completeness, and reasonability of the revised single items.

### Data analysis and item revision

For the data analysis, the Content Validity Index (CVI) was calculated for each item (I-CVI) [17]. The CVI scores were calculated as the proportion of raters who rated the item with an acceptable content validity (range 0–1.0). Lynn provides an orientation concerning the threshold values of the calculated I-CVI [17]. The CVI threshold value varies according to the number of experts with respect to their answers.

In this study, eight experts participated in the first round. However, due to missing answers in the second round, the CVI threshold values were adapted accordingly. In the case of eight complete answers, the threshold value was set at .88 (agreement of seven experts); for seven complete answers, the threshold value was set at .86 (agreement of six experts). The recommended threshold values were used as an indication for either exclusion or revision [19].

The judgment of relevance was the sole criterion to decide whether an item should be excluded in the first round. Items were revised if the judgment fell below threshold values for comprehensibility, reasonability, and completeness in the first round. If the revised items were judged to be unsatisfactory a second time, they were removed from the instrument. After the second round of expert ratings, the questionnaire was revised again.

The result of this step was version 2.

### Step 3: pre-test

Version 2 of the DemCare-Q was tested in a qualitative and a quantitative pre-test with future users. Therefore, two groups were constituted: a self-administered and an interviewer-administered group. For the self-administered group, an additional feedback questionnaire was provided to report difficulties in completing the questions.

### Study participants

Seven nursing homes were recruited to conduct the pre-test. In the self-administered group, 145 residents were assessed (using version 2 of the DemCare-Q) by registered nurses (RN) and certified nurse assistants (CNA) working in the participating institutions. To ensure confidentiality, no information on the assessing nurses was assessed.

Ten interviews were conducted in four of the participating nursing homes with seven RNs and three CNAs. The interviewed RNs had a mean work experience of seven years and had worked in the institution on average for three years. The CNAs had worked in nursing care for an average of 14 years and had worked in the institution for ten years.

### Testing procedures

The self-administered group was prepared to complete the questionnaire in the same way as it is planned for the data collection in the study mentioned (DemenzMonitor) [6]. One study coordinator at each nursing home received a one-day training at which the questionnaire was explained by members of the research team. The study coordinator was responsible for the entire data collection process. This person collected the data himself or designated and trained a staff member to perform the data collection.

In the interviewer-administered group, the questionnaire was completed in an interview that was conducted by one of two researchers (RP, KK). While the participant was answering the questions, various cognitive techniques (probing, thinking aloud, paraphrasing, confidence rating) were applied [20, 21]. “Information Retrieval Probes” asked the participants where they obtained the information to answer the questions; “Comprehension Probes” asked the participants to explain their understanding of terms and questions; and “Category Selection Probes” asked the participants to explain their answers. Additionally, the participants were asked to think aloud while reading the questions and selecting the answers to obtain insight into the answering processes. If terms were unclear, the participants were asked to paraphrase the term. Each interview was recorded with the permission of the respondents.

### Data analysis

The self-administered questionnaires were analyzed for inadequate answers and item non-response (i.e., missing answers) to demonstrate patterns of response behavior. The data on the evaluation interviews were analyzed using an adapted analysis scheme based on the Question Appraisal System (QAS-99) [22]. The analysis scheme focused on problems with comprehension of the questions and answers as well as problems with knowledge and information retrieval. One researcher (RP) listened to the records and documented problems according to the scheme. The responses from the feedback questionnaire were summarized and compared with the results of the evaluation interviews. If a problem was documented in the feedback questionnaires that did not appear in the evaluation interviews, it was added to the problem list.

Based on the results of the pre-test, the questionnaire was revised again (version 3).

## Results

### Structure of the DemCare-Q

The concept of “Dementia Care in Nursing Homes” was divided into seven topics, each containing a set of items. Each item has two parts: a lead-in question and a response set. Table 1 shows an overview of the three versions of the questionnaire.

**Table 1:** **Overview of the questionnaires of version 1, 2, and 3**

*Version*					*Version*			
1	2	3	Lead-in question	Response format	1	2	3	Response-set
**No.**	**No.**	**No.**						
***Assessments***								
1.	1.	/	Was an assessment conducted with a standardized instrument?	MR	✓	✓	/	Behavior
					✓	✓	/	Cognition
					✓	✓	/	Pain
					✓	✓	/	Depression
					/	✓	/	Quality of life
					/	✓	/	Mobility
					/	✓	/	Nutrition
					/	✓	/	Care dependency
					✓	✓	/	Other
2.	2.	/	How was the pain assessment (PA) conducted?	RO	✓	✓	/	Self-rated pain assessment
								Proxy-rated pain assessment
3.	3.	/	Which instrument was used for self-rated PA?*	RO	✓	✓	/	Numerical rating scale
								Visual analogue scale
								Verbal rating scale
								Smiley scale
								Face pain scale
								Other
4.	4.	/	Which instrument was used for proxy-rated PA?*	RO	✓	✓	/	BESD^©^
								BISAD^©^
								ECPA^©^
								ZOPA^©^
								Doloplus
								Other
/	/	6.	Was a PA conducted with a standardized instrument?	Y/N	/	/	✓	
/	/	7.	Which instrument was used for PA?*	RO	/	/	✓	Numerical rating scale
								Visual analogue scale
								Verbal rating scale
								Smiley scale
								Face pain scale
								BESD^©^
								BISAD^©^
								ECPA^©^
								ZOPA^©^
								Doloplus
								Self-developed
***Understanding diagnostics***								
5.	5.	1.	Has a case conference (CC) been held since the resident moved into the nursing home?	Y/N	✓	✓	✓	
6.	6.	2.	When was the last CC conducted?*	FT	✓	✓	✓	
/	6.1	2.1	Estimated period of time*	FS	/	✓	✓	During the last week
								Within the last four weeks
								Longer than four weeks ago
7.	7.	3.	Who took part in the last CC?*	MR	✓	✓	✓	Resident
					✓	✓	✓	Relative
					✓	✓	✓	Official legal guardian
					✓	✓	✓	Head nurse
					✓	✓	✓	Members of the nursing team
					✓	✓	✓	Other care staff
					✓	✓	✓	Physician
					✓	✓	✓	Therapeutic staff
					✓	✓	✓	Other
8.	8.	/	For what reason was the last CC conducted?*	MR	✓	✓	✓	Admission to nursing home
					✓	✓	✓	Health in general
					✓	✓	✓	Pain
/	/	4.	Why was the last CC conducted?*		✓	✓	✓	Problematic situations caused by challenging behavior
					✓	✓	✓	Hospital stay
					✓	✓	✓	Needs and wishes of the resident/relatives
					✓	✓	✓	Other
9.	9.		What was the content of the last CC?*	MR	✓	✓	✓	Reasons for challenging behavior
					✓	✓	✓	Biography
/	/	5.	What were you talking about in the last CC?*		✓	✓	✓	Decisions on care planning
					✓	✓	✓	Changes in medication
					✓	✓	✓	Discussion of previous care plans
					✓	✓	✓	Needs of the resident
					/	✓	✓	Daily activities
					/	✓	✓	Enhancement of competencies
					/	✓	✓	Relations of residents among each other
					✓	✓	✓	Other
***Reminiscence therapy***								
10.	10.	8.	Was the biography of the resident assessed?	Y/N	✓	✓	✓	
11.	11.	9.	Which topics were assessed?*	MR	✓	✓	✓	Important events in childhood- youth
					✓	✓	✓	Important events in adulthood
					✓	/	/	Professional life
					✓	✓	✓	Hobbies
					✓	✓	✓	Favorite food-drinks
					✓	✓	✓	Events of the day
					✓	✓	✓	Personality
					/	✓	✓	Relationships-social environment
					/	✓	✓	Habits
					✓	✓	✓	Other
12.	/	/	Who was involved in the biography assessment?*	MR	✓	/	/	Resident
					✓	/	/	Relatives-friends
					✓	/	/	Official legal guardian
					✓	/	/	Physician
					✓	/	/	Other
13.	12.	10.	Was anything added to the biography assessment after initial assessment?*	Y/N	✓	✓	✓	
***Multisensory stimulation***								
14.	/	/	Which of the following multisensory stimulation interventions have been applied, and if so, how often?					
			Aroma therapy	FS	✓	/	/	Daily/Weekly/Irregular
			Hand massage	FS	✓	/	/	
			Rhythmical massages	FS	✓	/	/	
			Snoezelen in a snoezelen room	FS	✓	/	/	
			Snoezelen in the resident’s room	FS	✓	/	/	
			Listening to relaxation music	FS	✓	/	/	
			Listening to individual preferred music	FS	✓	/	/	
/	13.	11.	Are there multisensory stimulation interventions applied?	Y/N	/	✓	✓	
/	14.	/	What kind of stimulation interventions?*	FT	/	✓	/	
/	/	12.		MR	/	/	✓	Aroma therapy
							✓	Sound therapy
							✓	Massages
							✓	Basal stimulation®
							✓	Snoezelen
							✓	Cuddling pets
							✓	Touching different materials
							✓	Other
***Validation therapy***								
15.	15.	13.	Is validation therapy applied?	Y/N	✓	✓	✓	
16.	/	/	How is validation therapy implemented?*	MR	✓	✓	✓	Integrated in daily communication/ Validating attitude
					✓	✓	✓	In single sessions with the resident
					✓	✓	✓	In group sessions
					/	✓	✓	As a crisis intervention
***Physical activities***								
17.	16.	15.	How often was the resident in the open air during the last week (e.g., on the balcony, in the garden, out for a walk)?	FS	✓	✓	✓	Several times a day/Daily/4-6 times a week/1-3 times a week/Not at all
18.	17.	/	Did the resident use any physical activities?	Y/N	✓	✓	✓	
/	/	16.	Did the resident use any physical activities offered during the last week?					
19.	18.	17.	Type of physical activity*	MR	✓	✓	✓	Gymnastics
					✓	✓	✓	Dance
					✓	✓	✓	Sports-games (e.g., bowling, ball games, games using a console (e.g., Wii))
					✓	✓	✓	Taking a walk
					/	/	✓	Physiotherapy
					/	/	✓	Occupational therapy
					✓	✓	✓	Other
20.	/	/	Reason for non-participation*	MR	✓	/	/	Not interested
					✓	/	/	Not able due to functional restraints
					✓	/	/	Not able due to cognitive restraints
					✓	/	/	Immobile
					✓	/	/	Other
21.	/	/	How often was the resident physically active at a minimum of 30 minutes at a stretch during the last week?	FS	✓	✓	✓	3x or more often/ 1-2x/ none
	19.	18.	How often was the resident physically active (e.g. participation on gymnastics or taking a walk) at a minimum of 30 minutes at a stretch during the last week?					
***Management of acute crisis intervention***								
22.	/	/	Has an acute psychiatric crisis occurred since admission that required nursing interventions?	Y/N	✓	✓	✓	
/	20.	/	Has an acute psychiatric crisis occurred during the last year that required nursing interventions?					
/	/	19.	Has an acute psychiatric crisis occurred during the last six months that required nursing interventions?					
/	21.	20.	Frequency of acute crisis during the last year during the last six months*	FS	/	✓	✓	1-2 times/ 3–4 times/ 5–6 times/more often
			Frequency of acute crisis during the last six months*					
23.	22.	21.	Applied interventions to manage the crisis*	MR	✓	✓	✓	Consultation with next of kin
					✓	✓	✓	Calming talk
					✓	✓	✓	Supporting the resident’s emotions
					✓	✓	✓	Protecting the resident from others
					✓	✓	✓	Consultation with a physician
					✓	✓	✓	Use of psychotropic medication
					✓	✓	✓	Use of physical restraints
					✓	✓	✓	Hospital admission
					/	✓	✓	Offering a possibility to reduce physical aggression
					✓	✓	✓	Other
**Total number of**								
**Lead-in questions**					**Responses**			
**23**	**22**	**21**			**81**	**79**	**78**	

MR –Multiple response RO-Response option FS-Frequency scale FT-Free text Y/N-Yes/No.

*Depending on previous question (conditional question).

### Revision of the questionnaire following expert ratings

After the first round of expert ratings, one question was deleted because the experts judged it as not relevant. Three questions and six response sets were revised due to low CVIs (Table 2). To improve comprehensibility, the revisions comprised rewording according to the comments of the experts and adding examples to clarify the meaning of terms. To improve the completeness of several response sets, new items were added or items were summarized and reworded. This process led to an improvement of I-CVIs after revision. Two response sets remained unsatisfactory after the revision and did not reach satisfactory values in the second round of judgment. Both were again revised for further testing. Another response set that did not improve content validity after revision (no. 20) was deleted.

**Table 2:** **Content validity index before and after expert ratings**

Version 1 question no.	Indicated problem	Category	Initial I-CVI’s ^1^	Revision	Final I-CVI’s ^2^
1.	Response set	Completeness	.14	Addition of 4 items	.86
		Reasonability	.75		1.0
11.	Response set	Completeness	.67	Addition of 2 items, deletion of 1 item, rewording of single items, addition of item examples	.86
		Reasonability	.56		.86
12.	Question	Relevance	.67	Deleted	/
14.	Response set	Completeness	.63	Summarization of items, revision of items	.43
		Reasonability	.38		.43
14. (Frequency)	Response set	Completeness	.75	Retained	/
16.	Question	Comprehensibility	.67	Reworded	1.0
	Response set	Completeness	.50	Addition of 1 item	1.0
		Reasonability	.56		1.0
20.	Response set	Reasonability	.67	Rewording and summarization of items	.71
		Completeness	.75		.86
21.	Question	Comprehensibility	.63	Addition of examples	1.0
22.	Question	Comprehensibility	.56	Adaption of reference period	.86
23.	Response set	Completeness	.56	Addition of one item	1.0

^1^Threshold value for content validity.88 (based on eight rater judgments).

^2^Threshold value for content validity .86 (based on seven rater judgments).

The number of lead-in questions and responses changed during the process of development and testing. In version 1, the questionnaire contained 23 lead-in questions with 81 responses. After the expert ratings, the questionnaire contained 22 lead-in questions with 79 responses.

### Results of the pre-test

The pre-test indicated that the participants had problems with 14 items. Comprehension problems were most common, occurring in seven questions and three response sets. The participants had also problems responding to five questions because they could not retrieve the necessary information. For five questions, these problems led to high item non-response. An overview of the indicated problems and the following revision is shown in Table 3.

**Table 3:** **Indicated problems during the pre-test of version 2 and revisions for version 3**

Version 2 question no.	Problem with	Indicated problem	Source	Revision for version 3
1.	Response	Comprehension (terms are too vague)	CI, FBQ	Deleted
		Response behavior	I-NR	
2.	Response	Comprehension (unclear term)	CI	Deleted
	Response	Response behavior	I-NR	
3./4.	Response	Comprehension (unclear term)	CI	Deleted
	Response	Information retrieval (missing knowledge)	CI	
	Response	Response behavior	I-NR	
5.	Question	Information retrieval (recall failure)	CI	Explanation added in the manual
	Question	Comprehension (unclear term)	FBQ	
	Response	Response behavior	I-NR	
8.	Question	Comprehension (wording)	CI	Reworded
9.	Question	Comprehension (wording)	CI	Reworded
11.	Response	Response behavior	I-NR	Retained
14.	Response	Information retrieval (missing knowledge)	CI	Development of categories
15.	Question	Comprehension (unclear term)	CI, FBQ	Explanation added in the manual
17.	Question	Comprehension (missing reference period)	CI	Addition of a reference period
18.	Question	Comprehension (unclear terms, items overlap)	CI	Explanation added in the manual
19.	Question	Information retrieval (missing knowledge)	CI	Retained
20.	Question	Comprehension (unclear term)	CI,FBQ	Explanation added in the manual
	Question	Information retrieval (recall failure)	CI	Reduction of reference period

CI-Cognitive interviews FBQ-Feedback questionnaire I-NR Item non-response.

### Comprehension

Comprehension problems occurred mainly due to a lack of clarity. The participants had problems understanding the meaning of the terms and questions due to problems with the wording or overcomplicated sentence syntax. Other response categories were too vague, and there were multiple ways to interpret them. The participants stated that the provided response categories did not match what they were doing in practice. Consequently, they had difficulty deciding what to choose. One question was unclear because the reference period was missing.

### Information retrieval

The participants indicated problems with information retrieval for questions when the information was not documented. Then, participants had difficulty responding because either they could not recall the information, or they did not have the information. The latter was the case, when interventions had been applied by another staff group.

### Response behavior

The indicated problems led partly to an inadequate response behavior, which was evident in a high rate of missing values in the dataset. The question whether assessments concerning different aspects of care had been performed was not answered in 6% (n = 8) of the 145 completed questionnaires. The question whether a self- or proxy-rated assessment was applied was not answered in 14% (n = 20) of the cases. The question which assessment instrument was used to assess pain was not answered in 22% of the questionnaires (n = 33). Problems with information retrieval concerning the occurrence of a case conference led to an 11% rate (n = 16) of item non-response.

### Revision of the instrument following the pre-test

As shown in Table 3, several revisions were made following the pre-test.

Four questions were deleted- and two new- simplified questions were added. To improve comprehension, two questions were reworded based on the suggestions made during the interviews. To enhance accuracy, reference periods in two questions were adapted. For one question, a new response set was developed based on the results of a free-text item in the previous version. For four questions with comprehension problems of the terms used, explanations were added to the questionnaire manual instead of revising the question and its response sets.

After the pre-test, the questionnaire was shortened to 21 lead-in questions with 78 responses.

## Discussion

The aim of this study was to develop and test a staff self-report questionnaire on the application of dementia-specific interventions in nursing homes, that is content valid and feasible for use in large-scale studies. The DemCare-Q assesses the application of the following recommended dementia-specific interventions: administration of a pain assessment, case conferences, reminiscence therapy, multisensory stimulation, validation, physical activities and the management of an acute psychiatric crisis.

### Evaluation of the application of dementia-specific interventions with the DemCare-Q

According to the national recommendation for the management of challenging behavior in people with dementia living in nursing homes, assessment of behavior, pain and cognition is suggested [5]. In the development process of the DemCare-Q, we were faced with the considerable difficulties of future users of the survey DemenzMonitor in answering the questions regarding the administration of behavior and cognition assessments. The nurses could not answer reliably if a standardized assessment was performed for each of the categories, and they had also difficulties in naming the instrument. The instrument from Wingenfeld et al. [12] collects information on behavior assessments: if a standardized assessment was performed, which instrument was used, which behavioral patterns were considered (e.g., agitation, depression, wandering) and which characteristics of the behavior were assessed (prevalence, frequency, special needs resulting from the behavior). They report difficulties similar to the ones in our study. The nurses had problems understanding the meaning of the term *‘assessment with the help of an instrument’.* This problem was solved by improving the manual for the instrument and intensifying the training of the nurses. Wingenfeld et al. also report nurses having difficulty retrieving information on which instrument was used for behavior assessment; this could be solved by adapting the nursing records from which the information could easily be retrieved. The results from the pilot study of Wingenfeld et al. [12] showed that behavior assessments were performed only in 5 of 45 participating nursing homes. One can conclude that behavior assessments are more an exception than the rule in German nursing homes and that the nurses were therefore not able to answer questions on behavior assessments. The same can be assumed for cognition assessments. Because the collection of data on the application of behavior and cognition assessments is relevant for the DemenzMonitor study, our questionnaire needs to be adapted for future data collections in this regard. We consider the addition of questions on behavior and cognition assessments to be reasonable, and this makes an adaption of the manual and training obligatory.

Regarding the assessment of information on case conferences and management of an acute psychiatric crisis, the initially developed questions worked out well in the expert evaluation and the pre-test. Therefore, only small adaptations were necessary. In version 3, we collect information on the time point, participants, reason for and content of the last case conference; occurrence and management strategies of an acute psychiatric crisis. To assess the application of reminiscence and validation therapy, the questions and responses that were developed and evaluated by the experts worked well in the pre-test, such that no changes were necessary. In version 3, the questionnaire collects information on whether a biography assessment was applied, whether information was added after the initial assessment and which biography topics were assessed. Regarding validation therapy, it assesses if and which form of validation therapy is provided. Regarding physical activity, the questionnaire collects information on the frequency and duration of different programs. Also here, the questions and answers that were confirmed by the experts worked well in the pre-test.

To the best of our knowledge, no existing instrument assesses comparable information on these dementia-specific nursing interventions that can be used in surveys.

### Critical reflection on the development and testing procedures

The measurement of nursing activities implies several challenges: they are multi-faceted and diverse with respect to the characteristics of their application. Although for some interventions it is rational to measure the time point of application and the duration and frequency (e.g., administration of assessment scales), this is not rational for other interventions, where other aspects are more important (e.g., validation). Every intervention has a different theoretical and rational base, which makes it unreasonable to develop a unitary measurement scale that can be applied to every intervention.

The development process of the DemCare-Q comprised different steps: literature review and analysis, expert ratings and a qualitative and quantitative pre-test with potential future users.

With the help of a literature review and a structured analysis of the literature, we were able to operationalize a first set of items. The analysis of the guidelines as well as the literature review showed that not all recommended interventions and their practical application were described properly in the reviewed material [16]. The lack of a concrete basis for the intervention components made the operationalization difficult. As a result, the interventions that were well described in the literature (e.g. case conferences) could be operationalized in detail and used to formulate sophisticated items in the questionnaire. Interventions that were not well described in the literature (e.g., validation) were operationalized on a more abstract level and contributed to significantly fewer items in the questionnaire.

The expert ratings led to a reduction of items in the revision and the calculation of CVIs made the improvement of content validity transparent. Moreover, the process sensitized us to which items could lead to difficulties when applying the questionnaire. Concerning the expert ratings, the approach from Lynn [17] was used, which has previously been applied in other studies to test content validity [23–25]. Crucial issues for testing content validity by an expert panel are the selection of the experts, the number of experts chosen, and the threshold values that indicate a need for revision or deletion. Because the soundness of the validation process is influenced by the inclusion criteria and utilization of the content experts [18], in this study, the requirements concerning qualification and experience were defined in advance. The selected experts had expertise in research on the provision of dementia care in German nursing homes or clinical expertise demonstrated by their work experience and qualifications. However, it was assumed that their expertise concerning the various aspects of the questionnaire varied. One strategy to account for this situation would be to define subsets of the experts [18]. We refrained from using this strategy because a higher number of experts are needed to form groups of more than five experts. Concerning the number of experts and the threshold values for the CVI, we followed the recommendations from Lynn [17] and recruited more than five experts to control for the possibility of chance agreement. For the analysis, we also refrained from analyzing the data in subsets because it was not obvious that there were significant differences in the ratings between practical experts and those from research.

The pre-test contributed to improvement of the content validity by revealing user understanding and response processes. Comprehension problems occurred mainly because of a lack of clarity of the terms or because the terms and words researchers used where unfamiliar to the nurses. Comprehension problems may also cause differences in the answering process, which, in turn, weakens the reliability of an instrument.

We identified dominant trends in the cognitive pre-test that were in line with the findings from the quantitative pre-test. In general, if problems with comprehension were apparent in more than one interview, this was also reflected by unsatisfactory response behavior in the self-administered group. Some problems occurred only in single interviews. This was mainly the case for interview partners who were not native German speakers but despite that speaking German fluently. It became obvious that these participants misunderstood the meaning of certain terms and definitions and therefore could not answer the questions precisely. Given the growing percentage of nurses with an immigrant background, this finding is important and must be considered in the planning of the data collection in future studies. If a problem with comprehension occurred for this reason, the research team decided to retain the term but added an explanation in the manual.

In summary, the pre-test uncovered problems of the questionnaire that could be solved by revision; but it also indicated the feasibility of the questionnaire. The distribution of the answers showed plausible results and, for the majority of items, the item-non-response rate was satisfactory.

These results should be discussed with regard to the methods applied and its possible constraints. For the cognitive pre-test, we decided to include a rather small group of interview participants. The literature recommends recruiting at least 30 participants for studies using cognitive techniques [26]. However, the authors of this recommendation note that a small sample size does not mean that the cognitive approach is deficient [27]. Because the purpose of the interviews was not a precise statistical estimation but rather the development of an understanding of cognitive processes, the variety of individuals included in the study was more essential than the number. Contrary to the method described by Fowler [20], who classifies questions as problematic if 15% or more of the responders had problems with the question, Willis [27] argues that problems in survey questions cannot be evaluated simply by counting the number of interviews in which a problem occurs. In our study, we included participants with different educational levels and a broad range of work experience. Due to the small number of interviews, we followed the recommendations of Willis [27] and refrained from calculating relative frequencies of problem indicators, but we discussed every problem that occurred. A potential limitation of the results of the pre-test is that the interviews were analyzed by only one researcher. To account for this limitation, each identified problem was discussed in the group. In case of inconsistency, the group listened to the records to verify the problem.

## Conclusions

One main challenge in assessing the prevalence of applied interventions in practice in a standardized manner lies mainly in developing questions and responses that are

- clear and precise, and
- presented in the language of the users.

If both conditions are unsatisfied, answers to the questions are either not given or not valid.

Operationalization requires a clear definition of the interventions and their components, which was often lacking for dementia care interventions. The method used here to form an initial set of items (literature review) did not yield to items that covered all aspects of dementia care. The difficulty of unclear terms, specifically terms used in multiple ways in practice, resulted in multiple revisions and even deletions of items that could not be replaced during the development process. For both reasons, the questionnaire needs to be revised with respect to missing aspects as well as better definition of problematic items such that they are understood and in line with practice.

With the help of the cognitive interviews, we developed a good understanding of what information can be reliably retrieved because it is documented in the residents’ records or a part of standardized procedures. For some items, we realized that respondents were uncertain of their answers, so that we suspect that these answers are not reliable. Therefore, investigation of inter-rater agreement is needed to ensure reliability and further revise the questionnaire. Currently, this is part of the actual measurement cycle of the DemenzMonitor 2014.

However, the study showed how the applied methods complement one another and ensure a continuous improvement process. The publication of this process enhances the transparency of questionnaire design and supports researchers in solving problems in developing questions to assess the application of interventions.
